# Capacitive and Conductometric Type Dual-Mode Relative Humidity Sensor Based on 5,10,15,20-tetra Phenyl Porphyrinato Nickel (II) (TPPNi)

**DOI:** 10.3390/polym13193336

**Published:** 2021-09-29

**Authors:** Rizwan Akram, Muhammad Yaseen, Zahid Farooq, Ayesha Rauf, Ziyad M. Almohaimeed, Muhammad Ikram, Qayyum Zafar

**Affiliations:** 1 Department of Electrical Engineering, College of Engineering, Qassim University, P.O. Box 6677, Buraydah 51452, Saudi Arabia; rizwanakram75@qec.edu.sa (R.A.); z.mohaimeed@qu.edu.sa (Z.M.A.); 2 Department of Chemistry, Division of Science & Technology, University of Education Lahore, Lahore 54000, Pakistan; m.yaseen@ue.edu.pk; 3 Department of Physics, Division of Science & Technology, University of Education Lahore, Lahore 54000, Pakistan; zahidv13@hotmail.com; 4 Department of Physics, University of Management and Technology, Lahore 54000, Pakistan; s2018139001@umt.edu.pk; 5 Solar Cell Applications Research Lab., Department of Physics, Government College University, Lahore 54000, Pakistan; dr.muhammadikram@gcu.edu.pk

**Keywords:** humidity sensing, capacitive and conductometric sensor, porous surface morphology, Grothus mechanism, response and recovery time

## Abstract

(1) Background: A quest for a highly sensitive and reliable humidity monitoring system for a diverse variety of applications is quite vital. Specifically, the ever-increasing demand of humidity sensors in applications ranging from agriculture to healthcare equipment (to cater the current demand of COVID-19 ventilation systems), calls for a selection of suitable humidity sensing material. (2) Methods: In the present study, the TPPNi macromolecule has been synthesized by using a microwave-assisted synthesis process. The layer structure of the fabricated humidity sensor (Al/TPPNi/Al) consists of pair of planar 120 nm thin aluminum (Al) electrodes (deposited by thermal evaporation) and ~160 nm facile spin-coated solution-processable organic TPPNi as an active layer between the ~40 µm electrode gap. (3) Results: Electrical properties (capacitance and impedance) of sensors were found to be substantially sensitive not only on relative humidity but also on the frequency of the input bias signal. The proposed sensor exhibits multimode (capacitive and conductometric) operation with significantly higher sensitivity ~146.17 pF/%RH at 500 Hz and 48.23 kΩ/%RH at 1 kHz. (4) Conclusions: The developed Al/TPPNi/Al surface type humidity sensor’s much-improved detecting properties along with reasonable dynamic range and response time suggest that it could be effective for continuous humidity monitoring in multi environmental applications.

## 1. Introduction

Effective and reliable humidity monitoring is of prime significance in an increasing number of industrial sectors such as in the chemical, electronics, pharmaceutical, agricultural, and HVAC (heating, ventilation, and air conditioning) sectors [1,2,3]. Particularly with the recent emergence of the internet of things (IoT) technology, humidity sensors are the utmost important components in developing state-of-the-art systems such as in smart farming, storage monitoring, healthcare equipment (CPAP machines and ventilators), and home automation [4,5,6,7]. Commercially available humidity monitoring devices typically resort to measurements of moisture-related changes in temperature, pressure, mass, or mechanical or electrical parameters of the active sensing material from which the moisture content can later be indicated [8,9]. However, the most ubiquitously utilized transduction techniques rely either on a variation in the conductivity or dielectric constant of the hygroscopic humidity sensing material [10].

A capacitive type sensor, for instance, consists of a pair of metallic electrodes separated by a dielectric material, whereas a conductometric type sensor consists of electrodes separated by a semi-conductive channel. As the relative humidity (%RH) of the environment increases, the dielectric constant of the capacitive sensor and conductivity of the conductometric sensor show a gradual increase corresponding to the increase in %RH [11]. The selection of humidity-sensitive active thin film is of prime importance while defining the superior performance of the sensor for electrical response (capacitive and conductometric) based sensing. Specifically, the materials selection for humidity sensing application is dictated by a couple of stringent requirements: superior porosity, hydrophilicity, and the inability of sensing layer to be dissolved in water [11].

Recently, conducting polymers based robust and ultra-sensitive sensors of biologically active compounds have been developed for their potential applications in biomedical diagnostics, the food and beverage industry, and environmental analysis [12,13]. Similarly, gas and volatile organic compound (VOC) sensors based on semiconducting metal oxides are also important for various safety and environmental control issues [14,15,16,17]. Likewise, the π-conjugated organic semiconductors (consisting of oligomeric/polymeric chain molecules) are a diverse set of materials that have been recently studied to develop cost-effective humidity sensors with superior sensitivity, reproducibility in response, and widespread bandwidth. The unique and exciting features of organic semiconductors (OSCs) such as water insolubility, mechanical flexibility, solution-process ability in organic solvents, biocompatibility, mixed ion and electron conductivity, open porous semi-conductive network with controlled pore size, and large specific surface area render them superior to their counterpart inorganic materials for humidity sensing applications [18,19,20]. In the quest of exploring OSCs, Porphyrins and their related families of molecules have been identified as the most promising class of macro heterocyclic compounds with unique ambient sensing properties [21,22,23]. Porphyrins and their derivatives (porphyrinoids) are of paramount R&D importance for their strong chemical and thermal stability and ability to coordinate with nearly all of the metal ions found in the periodic table. Interestingly, the molecular framework of porphyrin and their porphyrinoids provide a wide range of porphyrin-analyte interaction mechanisms, which include (a) the weak van der Waals forces, (b) π–π interactions, and (c) the coordination to the central metal ion, as well [1]. We believe that this fascinating feature of metalloporphyrins endows them with superior sensitivities to ambient humidity variation.

In the present study, we report a facile realization of surface type humidity sensor (Al/TPPNi/Al) based on 5,10,15,20-tetraphenylporphyrinatonickel(II). The fabricated sensor has been operated at multiple frequencies of input bias and its electrical response (capacitance and impedance) has been examined at varied ambient humidity levels. The purpose of the current research effort is to realize enhanced humidity sensing performance of the sensor compared to those already reported in the literature. 

## 2. Materials and Methods

### 2.1. Synthesis of TPPNi

The TPPNi macromolecule has been synthesized in two successive stages. The condensation of benzaldehyde and pyrrole yielded 5,10,15,20-tetraphenylporphyrin (TPP) in the first stage [24]. The detailed procedures involve the adsorption of a mixture of benzaldehyde (0.04 mole, 4.25 g) and pyrrole (0.04 mmole, 2.68 mg) over acidified silica gel (5.0 g) followed by 6 minutes of irradiation with 200 W microwave at 100 °C. The free base porphyrin (TPP) chemical was produced in a 24 percent yield after purification by column chromatography over silica gel with chloroform and n-hexane (2:1) as the eluent.

**Figure 1:**
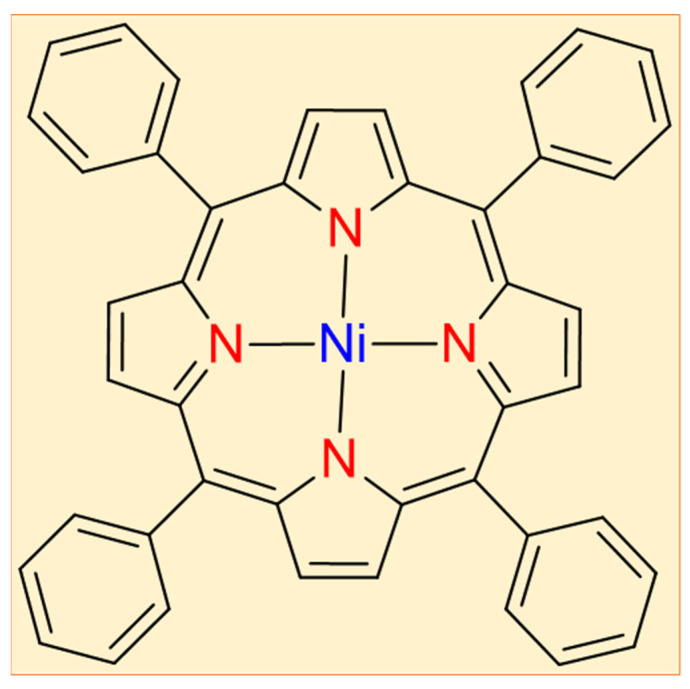
5,10,15,20-tetraphenylporphyrinatonickel(II) (TPPNi) chemical structure.

In the second step, porphyrin (0.04 mmole, 24.56 mg) and nickel acetate (1 mmole, 176.78 mg) after dissolving in a mixture of chloroform and methanol (10:1) have been adsorbed over silica gel. After drying, silica gel was microwave irradiated (250 W) for 15 minutes at 111 °C. The reaction mixture was then applied to the top of a silica column after cooling and eluted with a chloroform and n-hexane (1:4) combination. To obtain pure 5,10,15,20-tetraphenylporphyrinatonickel(II) in a good yield of 91%, the fast-moving band was collected, and the solvent was evaporated in vacuo (II). Figure 1 shows the chemical structure of the TPPNi molecule.

The chromatographic separation of compound and their purification depends chiefly upon the interaction between the solute in the mobile phase and the stationary phase. The exact conditions for the purification of any compound over the solid stationary phase by using a liquid mobile phase is determined by hit and trial method. Once the ratio of the solvents is determined, column chromatography process is performed with the same mobile and stationary phases. Reasonably, each compound has its own physical and chemical properties that eventually determine its distribution between the mobile and stationary phases during the chromatographic separation. In the case of TPP, the best separation on thin layer chromatography (TLC) plate was observed when the ratio of chloroform and n-hexane was (2:1), whereas in the case of TPPNi the aforementioned ratio was (1:4).

### 2.2. Fabrication of Humidity Sensor

**Figure 2:**
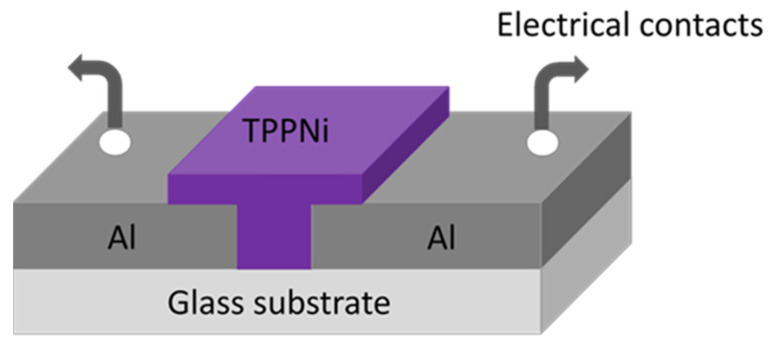
Schematic 3-D view of the Al/TPPNi/Al humidity sensor.

The humidity sensor has been fabricated in a surface-type configuration on a glass substrate using TPPNi as an active sensing layer. Regular soda lime microscopic glass slides (dimensions ~25 mm × 25 mm × 1 mm) have been used to function as the substrate for the fabrication of the device. The glass slides have been cleaned in two phases, at first using gentle rub via lint-free wipe and a cotton swab in soap-water. Later on, it was cleaned with a conventional cleaning procedure using Elmasonic E 30H ultrasonic cleaner (Elma Schmidbauer GmbH, Singen, Germany) for 10 min each with acetone, ethanol, and DI water followed by blown dried in a dust-free environment with a dry air stream. Through a shadow mask approach, an aluminum thin film with an average thickness of 120 nm was deposited on this glass substrate by a custom-designed physical vapor deposition (PVD) system at a rate of 0.2 nm/s. The PVD system is equipped with a single-stage rotary vane pump (Pfeiffer Vacuum GmbH, Berlin, Germany), (Pfeiffer, Hena 25, pumping speed ~25 m^3^/h) and a diffusion pump (Agilent Technologies, Santa Clara, CA, USA) (VHS-4, pumping speed ~750 L/s). Both pumps have been used to evacuate the chamber of the system to 5 × 10^−4^ mbarr (0.05 Pa). Shadow mask has been used to pattern spacing of ~40 µm between the pair of aluminum contact pads which have been defined to allow electrical connections of the humidity sensor. In order to deposit the active sensing layer of TPPNi, a 20 mg/mL TPPNi solution was prepared and stirred overnight by magnetic stirring. The solution was later passed through Polytetrafluoroethylene (PTFE) membrane filters of 0.45 µm pore size to filter it. Later, a 150 µL solution of TPPNi in chloroform was spin-coated to deposit a dielectric thin film covering the gap between the aluminum electrical contact pads. By this process, an average thickness ~160 nm of TPPNi thin film as a sensing layer has been observed by the Dektak profilometer. The cross-sectional schematic description of the Al/TPPNi/Al planar humidity sensor is given in Figure 2.

### 2.3. Sensor Testing Methodology

Physical Characterization: The UV-vis spectrum of the active thin film has been analyzed by the Jasco V-770 spectrophotometer. Nova NanoSEM 450 Field-Emission Scanning Electron Microscope (Field Electron and Ion Company FEI, Hillsboro, OR, USA) and Agilent Technologies 5500 Atomic Force Microscope (AFM) (Agilent Technologies, Santa Clara, CA, USA) were used to analyze the surface morphology of the active sensing layer. The structural properties of the active sensing layer have been investigated by studying X-ray Diffraction (XRD) pattern by using Shimadzu 7000 Diffractometer (Shimadzu Corporation, Kyoto, Japan) functioning with Cu Kα_1_ radiation (λ = 0.15406 nm) generated at 30 kV and 30 mA with a scan rate of 2° min^−1^ for 2θ values between 10° and 80°.

Electrical Characterization: The experimental setup used for sensor testing is a laboratory assembled (hermetically sealed) chamber. The humidity within the chamber has been controlled by dry and humid-air flow, routed through inlet and outlet regulatory valves. The reference levels (relative humidity, ambient temperature) inside the controlled environmental chamber have been effectively monitored by Pro’s Kit MT 4014 commercial Thermo-hygrometer (Prokit’s Industries, Taipei, Taiwan) with a resolution of ~0.1% RH and ~0.1 °C).

**Figure 3:**
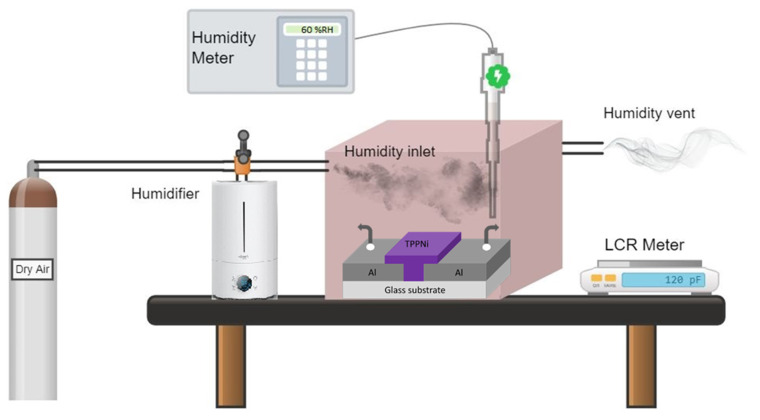
General arrangement of the testing setup used for characterization of Al/TPPNi/Al humidity sensor.

The proposed Al/TPPNi/Al sensor was characterized for its electrical characteristics (by exposing it to various humidity levels) with a high quality (measurement accuracy 0.1%) APPLENT AT2816B LCR Meter (Applent Instruments Inc, Jiangsu, China). Furthermore, the electrical response of the sensor was recorded at four distinct frequencies of the input signal (500 Hz, 1 kHz, 10 kHz, and 100 kHz), while the applied bias (V_rms_) was kept constant at 1.0 V. It is noteworthy that the high precision APPLENT AT2816B LCR Meter provides simultaneous measurement of both capacitance and impedance at all test frequencies, in a single cycle of experiment wherein the step input changes the range of 45% to 85% relative humidity is of course identical. It is also pertinent to mention that humidity is a relative function of temperature. At different temperatures, the humidity saturation value of the same space is different, and hence the relative humidity value is also different. All the experiments in the present study have been performed at 25 ± 0.5 °C. The general layout of the testing setup constructed for the calibration of humidity sensors is shown in Figure 3.

## 3. Results and Discussion

### 3.1. Physical Characterization of TPPNi Active Layer 

**Figure 4:**
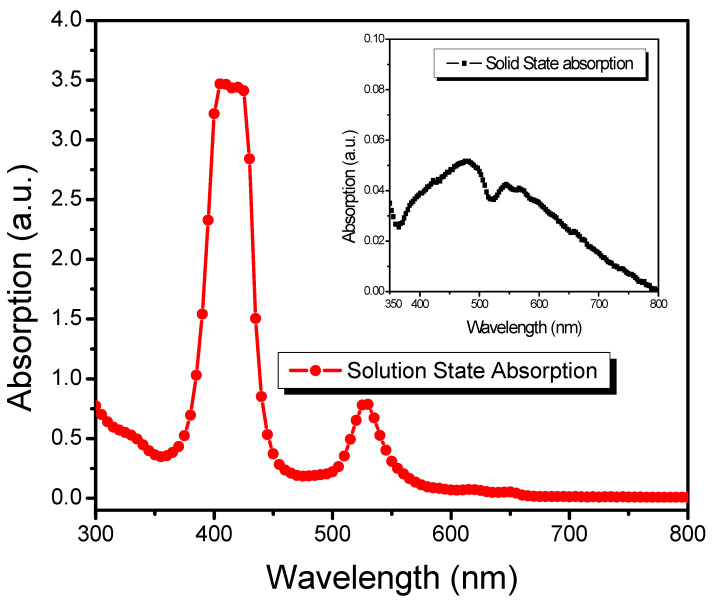
UV-vis absorption spectrum of TPPNi in solution state and (inset) solid-state (spin-coated thin film).

Optical Study of TPPNi: The optical properties of the TPPNi have been investigated using UV–vis absorption spectroscopy (wavelength range ~300–800 nm) in solution as well as solid-state, as shown in Figure 4 and Figure 4 (inset), respectively. 

Porphyrins have two electronic transitions in the visible domain of the electromagnetic spectrum: a Soret band at 350–500 nm and Q-bands around 500–700 nm with typically one order of magnitude lower intensity [25]. The UV–vis absorption spectrum of the TPPNi solution (in chloroform) displayed the characteristic Soret band between 355 and 465 nm, with a maximum absorption peak at 415 nm, which is attributed to the π–π* transition from the ground state (S_0_) to the second-lowest singlet state (S_2_). On the other hand, the broadband at 500–575 nm with peak absorption at 525 nm is due to π–π* electron transition from the ground state (S_0_) to the lowest excited singlet state (S_1_). Quite interestingly, albeit the absorption spectra of TPPNi solution (in chloroform) and thin film (prepared by spin coating its solution in chloroform on pre-cleaned glass substrates) are somewhat similar; however, there is a significant difference in the shape of the Soret band of both spectra. Specifically, in the solution state, TPPNi macromolecule exhibited a narrow Soret band; whereas, the Soret band has become substantially broader in the solid-state spectrum. In addition, the characteristic Soret band peak has been observed to be redshifted significantly. The observed results may be due to the aggregates formation in thin film, that ultimately results in an increased π–π interaction, as reported by some other studies [26,27,28]. 

**Figure 5:**
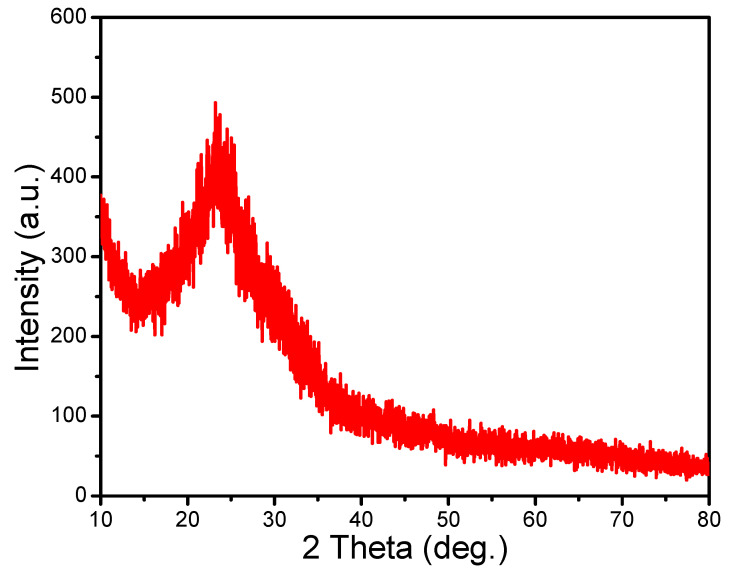
XRD diffractogram of TPPNi thin film.

Structural study of TPPNi: The crystalline structure of the TPPNi semiconducting layer has been analyzed by X-ray Diffraction (XRD) pattern, which displays diffraction intensity as a function of 2θ (as shown in Figure 5). Typically, the existence of a amorphous solid form can be confirmed by detecting the absence of the distinct XRD peaks, which are envisaged to be the characteristic of crystalline order [29]. The appearance of a general “halo” pattern at 2θ–23.5° may further point towards the occurrence of amorphous, glassy, or disordered material. 

**Figure 6:**
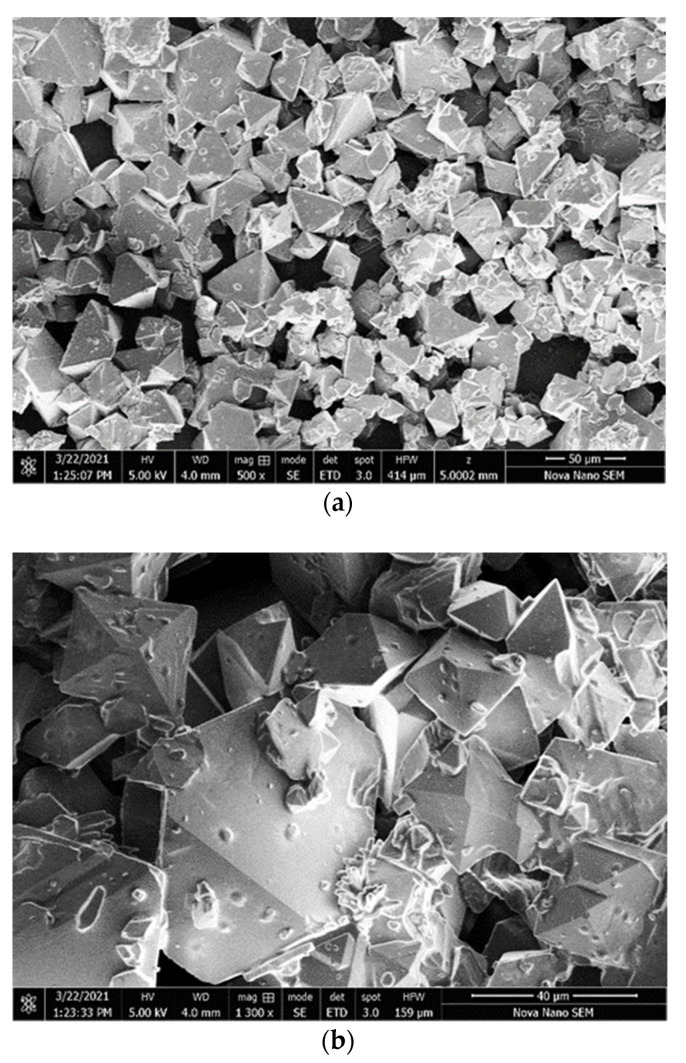
FESEM micrographs (surface view) of TPPNi active humidity sensing layer at (**a**) 500 and (**b**) 1.3k magnification scales.

Surface morphology of TPPNi thin film: field emission scanning electron microscopy (FESEM) has been used to characterize the surface morphology of a pristine TPPNi thin film. The FESEM micrographs (Figure 6a,b) depict the TPPNi thin film at different magnification scales (500 and 1.3k, respectively). It may be clearly observed that the humidity sensing TPPNi layer comprises essentially of micro-pyramidal shaped structures (decorated with inhomogeneous, irregular shaped sub-micron particles). In addition, the internal structure of the sensing layer contains a fine network of voids/pores resulting in a “sponge-like” structure. In fact, the porous morphological characteristic appears to be intrinsic for porphyrin-sponges which is a general name for a variety of phenyl-meso-exchanged metalloporphyrin analogues [30].

The microporous structure of the active thin film is envisaged to be vital for superior humidity sensing ability since it allows a stronger interaction between the analyte (water molecules) and the sensing layer. Hence, we believe that TPPNi is an ideal template for humidity sensing by virtue of bulk porosity and essential void spaces (between microstructures), which may assist the efficient humidity circulation through the bulk. Further, the irregular-shaped sub-micron particles embedded on the pyramid-shaped structures are also believed to provide a larger specific area for improved humidity adsorption.

**Figure 7:**
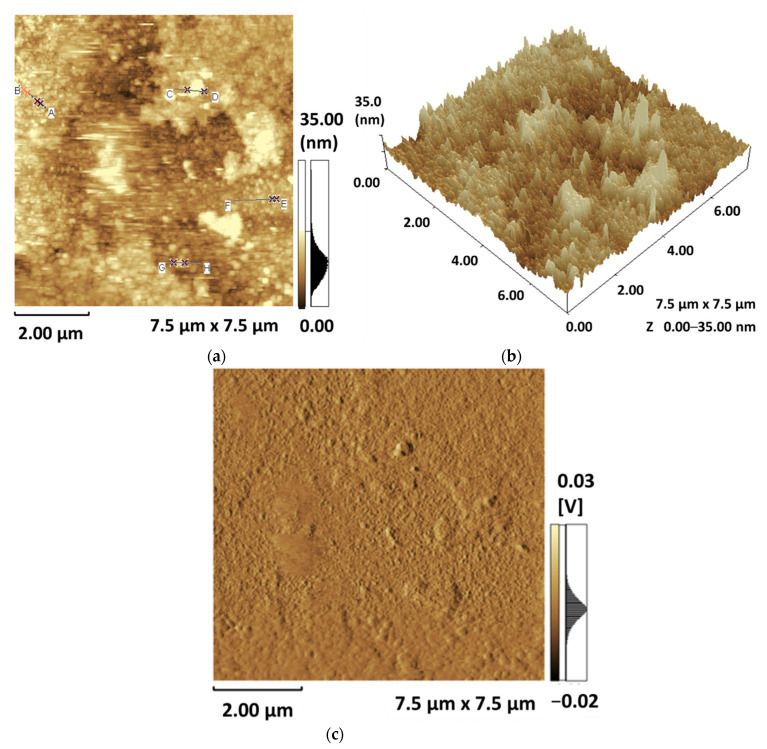

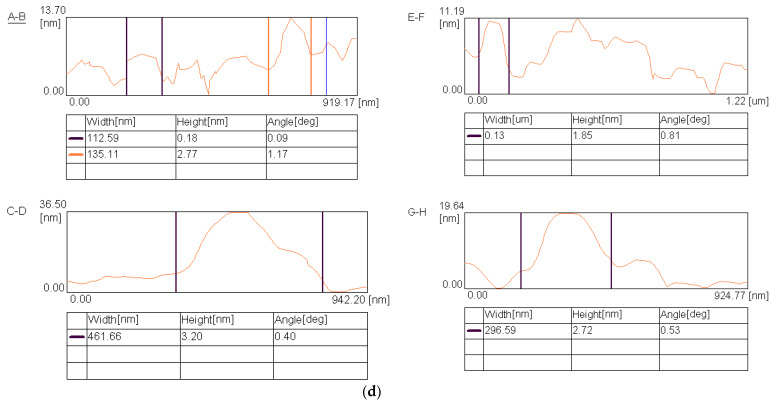
(**a**) 2D and (**b**) 3D AFM images of TPPNi thin film in contact mode, (**c**) 2D deflection mode AFM image and (**d**) cross-sectional surface profile of pristine TPPNi thin film.

To supplement the aforementioned experimental results, the morphology of the pristine TPPNi thin film has also been studied via atomic force microscope (AFM). Figure 7a–c depict the two, three-dimensional contact and 2D deflection mode AFM images of the spin coated pristine TPPNi thin film, respectively, with an examination area of 7.5 μm × 7.5 μm. The symbols “x” in Figure 7a, represent the characteristic points on the surface of the active sensing layer which may further be related with the colored vertical lines in Figure 7d. In A–B section of Figure 7a, two color-lines have been used to help probe morphology of two linear segments of active sensing layer, which may be related to two different vertical colored lines in A–B section of Figure 7d. 

Admittedly, the 2-D topographic AFM scan (Figure 7a) is significantly distorted. It is well-understood that the quality of AFM images depends greatly on operation, tip state and the hardness of sample surface [31]. In contact mode, AFM is generally operated using via cantilever tip that essentially maintains contact with the sample’s surface at all times. The force applied to the sample sometimes leads to poor images and distortion of soft samples (such as organic semiconductor thin film) by the tip due to capillary forces when imaging in air [32]. Hence, typically for surface morphology investigation of (very) rough surfaces, the 3D surface measurements (Figure 7b) are preferred over the top-view projections [33]. Figure 7d portrays the section analysis at four distinct randomly selected locations (pre-specified in Figure 7a). It may be clearly observed that the surface of the sensing layer is rough and exhibits positive skewness (i.e., the surface exhibits mainly peaks and asperities). The prominent high surface roughness in TPPNi humidity sensing film gives a significant rise to surface-to-volume ratio [34], which is ultimately expected to yield higher sensitivity of the humidity sensing device.

### 3.2. Electrical Characterization of Humidity Sensor

Humidity sensing performance study of TPPNi Generally, humidity influences a wide variety of physical, chemical and biological processes, and these effects can later be exploited to estimate variation in varied humidity levels [35]. When operated in capacitive mode, the fabricated humidity sensor utilizes the TPPNi sensing layer as a dielectric layer. The sensing layer adsorbs and desorbs the water molecules in proportion to the ambient relative humidity during its capacitive mode of operation. The area of the aluminum electrodes (*A*), inter-electrodes gap (*d*) and the dielectric permittivity constant (*ε_r_*) of the TPPNi dielectric material influence the capacitance of the fabricated device (represented mathematically in Equation (1) [36].

(1) $$C=\frac{\varepsilon_{0}\varepsilon_{r}A}{d}$$

where “*C*” is the capacitance of the fabricated device and “*ε*_0_” represents the dielectric permittivity of air.

Interestingly for efficient detection of chemical analytes/gas molecules via capacitive-sensing mechanism, the molecules of interest must induce a remarkable change either in *ε_r_*, *A*, or *d* (however for the case of the humidity sensor, changes in electrode area (*A*) or electrode separation (*d*) are pretty uncommon). The dielectric permittivity of vacuum is exactly 1, and that of notable gases is within 1% of unity [37]. Reasonably, the relative permittivity of most chemical analytes/inorganic gases are significantly smaller than water molecules (~80) (see Table 1). The changes in dielectric constant due to the interaction between active sensing layer and notable chemical analytes/gases molecules is therefore highly unexpected [38].

Dielectric permittivity of the active humidity sensing layer is triggered by polarization in the TPPNi layer (humid and desiccated). Typically, there are four mechanisms i.e., dipolar, ionic, space charge, or electronic, which may contribute towards polarizability of the active layer [40]. The Clausius–Mosotti equation defines the relationship between the dielectric constant (*ε_r_*) and polarizability (*α_d_*) as given in Equation (2) [41], where, *N_d_* and *α_d_* represent number density of molecules and polarizability in sensing layer in dry condition.

(2) εr=((1 + 2Ndαd)3ε0(1 − Ndαd)3ε0)

whereas Equation (3) describes the relationship between dielectric constant and capacitance [42].

(3) CSC0=(εwetεdry)n=((1 + 2Nwαw)3ε0((1 − Nwαw)3ε0)εdry)n

where, *N_d_* and *α_d_* represent number density of molecules and polarizability in sensing layer in wet/humid condition. Here *ε_dry_* and *ε_wet_* are the relative dielectric constants for the desiccated and humid active sensing layer, respectively, and “*n*” is the dielectric morphology related factor. Generally, the dielectric permittivity of desiccated organic semiconductor layer is ~5 which is considerably smaller than that of water ~80 [43]. Naturally, with the continuing adsorption of water molecules by the TPPNi thin layer, the dielectric permittivity of the humid sensing layer varies significantly [44].

**Figure 8:**
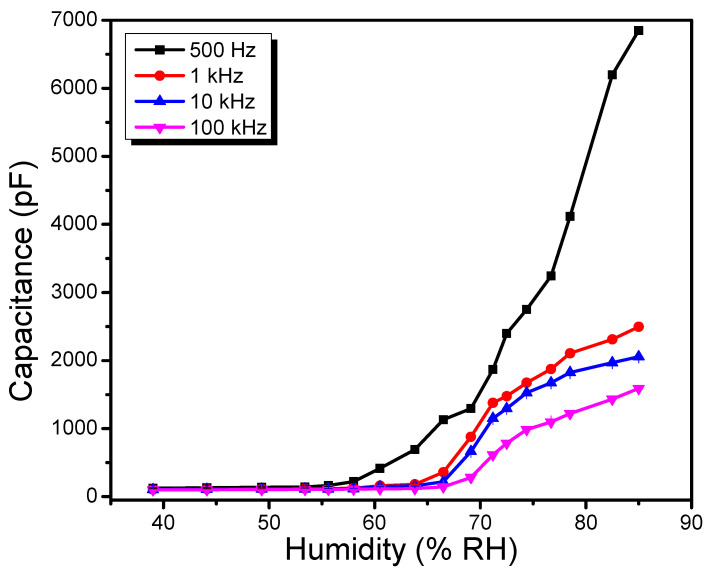
Effect of test frequencies on capacitance vs. %RH characteristics of Al/TPPNi/Al humidity sensor (error bar is too small to be visible on linear scale).

Figure 8 depicts the capacitance-relative humidity response of the fabricated humidity sensor for a range of 39 to 85%RH measured at four distinct frequencies (500 Hz, 1 kHz, 10 kHz and 100 kHz) of the AC test signal. In general, for all test frequencies, the capacitance of the fabricated device displays a monotonous nonlinear increase as a function of %RH. Moreover, this nonlinear response can be correlated to the prolonged relaxation period of the dipole moments of adsorbed water molecules [45]. In comparison to high test frequencies (1, 10, and 100 kHz), the influence of %RH variation on the capacitance was shown to be larger at low operating frequency (500 Hz). The sensor’s capacitance has shown an increase by 54.36 times in magnitude at test frequency ~500 Hz with an increase in %RH from 39 to 85%, as shown in Figure 8. A decrease in the capacitance change has been observed at higher frequencies, precisely 23.65, 19.58, and 15.77 times, for 1 kHz, 10 kHz, and 100 kHz, respectively. The sensitivity of the fabricated device towards ambient humidity has been measured to be 146.17, 51.94, 42.41, and 32.35 pF/%RH at four distinct frequencies of the AC test signal. This is very well correlated with the formerly established fact by E. Pinottie et al. that based on the low intrinsic mobility of organic semiconductors, in some cases, the charge carrier cannot follow the rapid change in the applied electric field due to applied test signal at higher frequencies [46]. As a result, the polarization mechanism becomes less effective, and the dielectric permittivity of the active layer decreases at higher frequencies [47,48].

The capacitance variation in the 39–58%RH range is not noticeable, as seen in Figure 8, due to the very well-known fact that the coverage of water molecules on the active sensing layer is not noticeable at low ambient humidity levels. Primarily, water molecules are chemisorbed (in the form of a monolayer) on the sensing thin film by virtue of the electron vacancies on the surface [47]. On the chemisorbed water layer template, several physiosorbed water molecular layers continue to accumulate as moisture levels rise [49]. Additional water adsorbed molecules strengthen the polarization and significantly increase the capacitance of the sensor [50]. Thus, in a range of 58–85%RH, a steady increase in capacity is conveniently observed.

For the ageing process, the sensor has been placed in ambient environment at room temperature for two months. The sensor has later been tested again under different %RH levels for two round tests. During the test, the sensor showed good stability, consistency and repeatability. Specifically, the sensor showed an average of ~3.42% decrease in capacitive response at 500 Hz after the ageing process. The capacitance of the fabricated humidity sensor has also shown reasonably good stability with the changes in temperature until ~55 °C. However, with further increase in the temperature from (55 °C–80 °C), the capacitance of the fabricated sensor exhibited an upsurge in its value. Specifically, the capacitance increase has been observed to be ~1.3 times of the initial value when the temperature was gradually increased from 55 °C–80 °C.

**Figure 9:**
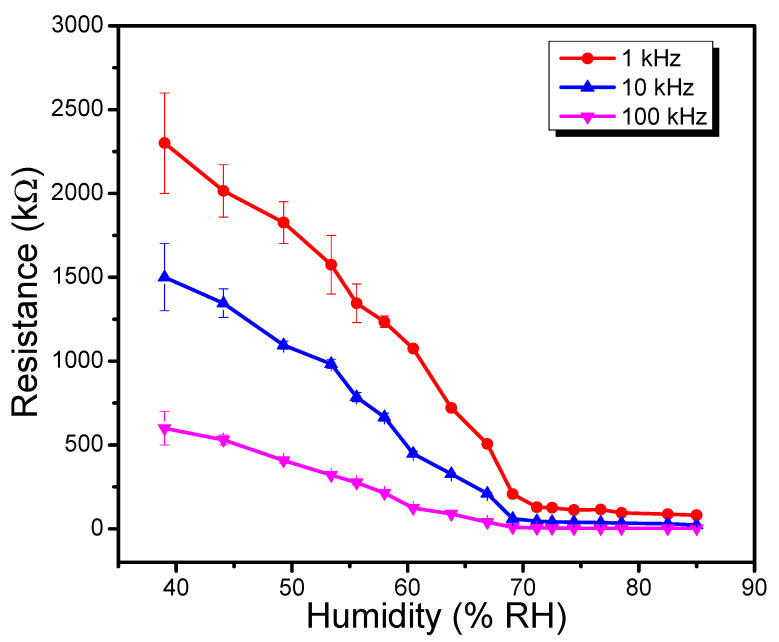
Effect of test frequencies on impedance vs. %RH characteristics of Al/TPPNi/Al humidity sensor.

Interestingly the OSCs provide a technological attractive charge transport property that is significantly modulated with ambient conditions, in particular humidity. The influence of ambient relative humidity, in the 39–85%RH range, on the impedance of the fabricated sensor for three test frequencies (1 kHz, 10 kHz, and 100 kHz) is depicted in Figure 9. It may be conveniently observed that for all test frequencies, impedance of the sensor exhibits a similar trend (i.e., decrease in magnitude with the upsurge in ambient relative humidity). At 1 kHz test frequency, an electrical impedance change of 28.32 times was detected at 85 percent RH compared to 39%RH, resulting in a 48.23 kΩ/ percent RH sensitivity. Similarly, the sensitivity at higher frequencies such as 10 kHz and 100 kHz sensitivity of 32.11 kΩ/%RH and 13.00 kΩ/%RH has been recorded. The aforementioned results prove that TPPNi semiconductor-based humidity sensor can effectively function in the dual (capacitive and conductometric) mode for ambient relative humidity monitoring.

The operating mechanism of impedance-type sensors may be described with the help of the Grothus mechanism. At low %RH range, primarily immobile chemisorbed water molecules layer is formed on the surface of TPPNi thin film, and the conduction of the active layer at this stage is mainly by virtue of intrinsic electrons only [51]. Furthermore, as the %RH level rises, layers of multi-physiosorbed water molecules are adsorbed on the active sensing layer. These physiosorbed layers exhibit liquid-like behavior and swiftly decompose into hydronium ions (H_3_O)^+^ as charge carriers, as described in chemical Equation (4). Therefore, the conductivity of the semiconductor thin film at higher %RH is now dictated by the ionic conduction [52]. In bulk, hydronium ion releases hydrogen ion (H^+^) to its neighboring water molecule, and the chain reaction continues. The effective proton hopping between neighboring molecules in physiosorbed H_2_O molecules layers considerably reduces the electrical impedance of the TPPNi sensing layer [53].

(4) $$H_{2}O+H_{2}O\;\overset{}{\Leftrightarrow }H_{3}O^{+}+HO^{-}$$

**Figure 10:**
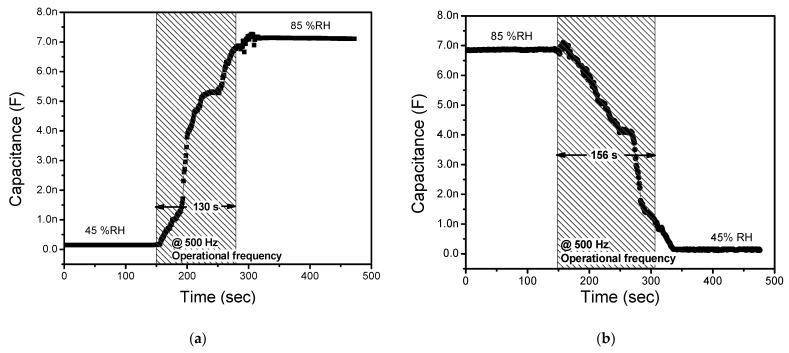
(**a**) Response and (**b**) reset time measurement of the proposed humidity sensor at 500 Hz test frequency.

When analyzing the sensor’s performance, the response time or the recovery/reset time is a critical parameter of interest. It is computed during the humidification/desiccation cycle of the humidity sensor’s dynamic curve [54]. The sensor’s temporal capacitive responses to step-change in ambient relative humidity levels are depicted in Figure 10a,b. As shown in Figure 10a, the sensor in capacitive mode shows a stable baseline initially when measured at 45%RH, and consequently, with step input in %RH from 45% to 85%, the average response time has been evaluated to be ~130 s. Similarly, the reset time in the capacitive mode of operation has been recorded to be 156 s, as shown in Figure 10b. The effective diffusivity of water molecules in the active sensing layer can be securely attributed to the constructed humidity sensor’s considerably slow response/recovery time. 

Table 2 compares the proposed TPPNi-based capacitive and conductometric humidity sensor to previously reported sensors in terms of critical performance metrics. Admittedly the proposed humidity sensor is a little inefficient in terms of response/reset time, the proposed sensor outperforms others in terms of sensitivity. It is expected that by selecting the right doping material (such as metal oxide nanostructures), the sensitivity will be improved, and the response time will be significantly reduced. The impact of doping and other geometrical parameters is being investigated and will be reported later.

## 4. Conclusions

Fabrication and characterization of TPPNi thin films for their use as surface type humidity sensors have been studied for the TPPNi synthesized by microwave-assisted method. An optical study is enabled to visualize the reason for broadening in Soret band absorption spectra and observed redshift in the peak absorption values for solid-state TPPNi in comparison with solution state TPPNi. The observed “halo” pattern in XRD structural characterization has clearly demonstrated that the fabricated thin films possess amorphous, glassy, or disordered structures. Furthermore, FESEM investigation has confirmed that TPPNi thin films comprise essentially of micro-pyramidal shaped structures, which is foresighted to be useful to increase the specific area for humidity absorption. The surface morphological study has also shown that the interior volume of the active sensing layers has fine pores/voids, which are speculated to be the main admittance sites for humidity and facilitates the adsorption kinetics of water inside the active sensing film. 

By registering AC capacitance and impedance, the feasibility of the recommended active layer for humidity sensing to differentiate between varying %RH levels has been demonstrated. With an increase of %RH level from 39 to 85 percent, the amount of capacitance and the impedance value has changed 54.36 times at 500 Hz and 28.32 times at 1 kHz. This incremental variation of capacitance is expected due to the high difference in dielectric permittivity constants of water and TPPNi thin film. The pronounced conductivity at the high order of humidity levels may be the source of the drop in the value of sensor’s impedance at raised %RH levels. The sensitivity of the fabricated devices towards ambient humidity has been measured to be 146.17 pF/%RH and 48.23 kΩ/%RH for capacitance and Impedance measured at 500 Hz and 1 kHz, respectively. The observed increase in the sensitivity compared to previously published noteworthy humidity sensors can be correlated to prominent high surface roughness in TPPNi thin films, which causes the high surface-to-volume ratio. In comparison with published set of humidity sensors, it has been shown that TPPNi semiconductor-based humidity sensor can effectively function quasi linearly in dual (capacitive as well as conductometric) mode for ambient relative humidity monitoring with superior sensitivity with a compromise in response recovery/reset time. 

## Figures and Tables

**Table 1:** Comparison of dielectric permittivity of notable gases/chemical analytes (Source: [37] and [39]).

Notable Gases/Chemical Analytes	Dielectric Permittivity (at 20 °C and 10^6^ Hz)
Helium (He)	1.000065
Hydrogen (N_2_)	1.000272
Oxygen (O_2_)	1.000494
Carbon dioxide (CO_2_)	1.000922
Ethylene (C_2_H_4_)	1.00134
Acetylene (C_2_H_2_)	1.00124
Ethane(C_2_H_6_)	1.0014

**Table 2:** Comparison of humidity sensors based on key performance parameters.

Material	Mode of Operation	Sensitivity	Bandwidth	Response/Reset Time
DMBHPET [55]	Capacitive	0.007 pF/%RH	30–80%RH	10, 15 s
Polyimide polymer [56]	Capacitive	22.29 pF/%RH	20–90%RH	25 s
Methyl-red [57]	Capacitive	16.92 pF/%RH	30–95%RH	~10 s each
ZnO-SnO_2_ composite thin film [58]	Conductometric	8.6 kΩ/%RH	32–92%RH	17, 65 s
Interpenetrating Polymer Network (IPN) thin films polyaniline/PVA [59]	Conductometric	12.6 kΩ/%RH	30–85%RH	-
Porous polyetherimide (PEI) polymer	Capacitive	0.38 pF/% RH	15–80%RH	
TPPNi	Capacitive and Conductometric	146.17 pF/%RH @ 500 Hz 48.23 kΩ/%RH @ 1 kHz	39–85%RH	130, 156 s

## Data Availability

All data has already been provided in the form of ORIGIN plots. Further requested data can be made available upon publisher’s request.
